# Single-Cell RNA Sequencing for the Detection of Clonotypic V(D)J Rearrangements in Multiple Myeloma

**DOI:** 10.3390/ijms232415691

**Published:** 2022-12-10

**Authors:** Antonio Matera, Alessio Marella, Akihiro Maeda, Matteo C. Da Vià, Francesca Lazzaroni, Sonia Fabris, Stefania Pioggia, Laura Porretti, Federico Colombo, Federica Torricelli, Antonino Neri, Elisa Taiana, Giuseppina Fabbiano, Valentina Traini, Elisa Genuardi, Daniela Drandi, Niccolò Bolli, Marta Lionetti

**Affiliations:** 1 Department of Oncology and Hemato-Oncology, University of Milan, 20122 Milan, Italy; 2 Hematology Unit, Fondazione IRCCS Ca’ Granda Ospedale Maggiore Policlinico, 20122 Milan, Italy; 3 Laboratory of Translational Research, Azienda USL-IRCCS di Reggio Emilia, 42123 Reggio Emilia, Italy; 4 Scientific Directorate, Azienda USL-IRCCS di Reggio Emilia, 42123 Reggio Emilia, Italy; 5 Department of Molecular Biotechnologies and Health Sciences, University of Torino, 10124 Torino, Italy

**Keywords:** multiple myeloma, single-cell RNA sequencing, clonal biomarker, V(D)J rearrangement

## Abstract

Multiple myeloma (MM) has a highly heterogeneous genetic background, which complicates its molecular tracking over time. Nevertheless, each MM patient’s malignant plasma cells (PCs) share unique V(D)J rearranged sequences at immunoglobulin loci, which represent ideal disease biomarkers. Because the tumor-specific V(D)J sequence is highly expressed in bulk RNA in MM patients, we wondered whether it can be identified by single-cell RNA sequencing (scRNA-seq). To this end we analyzed CD138^+^ cells purified from bone marrow aspirates of 19 samples with PC dyscrasias by both a standard method based on bulk DNA and by an implementation of the standard 10x Genomics protocol to detect expressed V(D)J sequences. A dominant clonotype was easily identified in each sample, accounting on average for 83.65% of V(D)J-rearranged cells. Compared with standard methods, scRNA-seq analysis proved highly concordant and even more effective in identifying clonal productive rearrangements, by-passing limitations related to the misannealing of consensus primers in hypermutated regions. We next validated its accuracy to track 5 clonal cells with absolute sensitivity in a virtual sample containing 3180 polyclonal cells. This shows that single-cell V(D)J analysis may be used to find rare clonal cells, laying the foundations for functional single-cell dissection of minimal residual disease.

## 1. Introduction

Multiple myeloma (MM) is a malignant proliferation of antibody-secreting bone marrow (BM) plasma cells (PCs) that accounts for slightly more than 17% of all hematological malignancies in the United States [1]. It is characterized by a highly heterogeneous genetic background and clinical course, and remains an incurable disease [2,3]. Mutations and copy number changes are not stable during the disease course from asymptomatic stages [4,5], newly diagnosed myeloma [6], and relapsed-refractory myeloma [7,8]. The clonal B cell origin of MM ensures that all malignant PCs share the same immunoglobulin (Ig) heavy and light chain variable regions. Due to the great diversity introduced during formation of the mature Ig gene through V(D)J recombination, junctional insertions/deletions and somatic hypermutation, it is nearly impossible that independent B cell clones share identical variable regions. The uniqueness of these tumor-specific sequences, along with their stability over time despite differential clonal evolution, make them ideal biomarkers for minimal residual disease (MRD) monitoring [9].

In recent years, the evaluation of the transcriptome of individual cells has become possible thanks to the development of single-cell RNA sequencing (scRNA-seq), a powerful technology exploited by several commercial platforms, all of which forecast single-cell isolation before capturing of RNA molecules, reverse transcription, cDNA amplification, library preparation and next-generation sequencing (NGS). ScRNA-seq is able to dissect cell-to-cell variation in tumors and microenvironments, and therefore its application has the potential to provide the greatest insights in cell populations characterized by high heterogeneity, such as the malignant PC clone in MM. It is therefore natural that great efforts of scRNA-seq in MM take place immediately, making it possible to obtain a detailed molecular characterization of tumor and immune cells in symptomatic and asymptomatic patients [10,11,12,13]. Because the tumor-specific V(D)J sequence is highly expressed in bulk RNA in MM patients [14], the question is whether it can also be identified in single-cells and with what accuracy as compared to clinical grade V(D)J diagnostics based on consensus primers and a DNA template. This would allow a whole new field of study based on single-cell analysis of MRD positive (MRD+) cells.

Here, we evaluated the efficacy of scRNA-seq in identifying the MM-specific V(D)J rearrangement by means of an implementation of the standard 10x Genomics protocol. The output thus generated from single BM PCs from 19 patients’ samples was then compared with the sequences of the rearrangements determined using a standard method based on the EuroClonality-NGS Working Group standard operating procedure applied on bulk BM PC DNA [15] and submitting data to the Vidjil web platform for analysis of high-throughput immune repertoire sequencing [16].

## 2. Results

**Figure 1:**
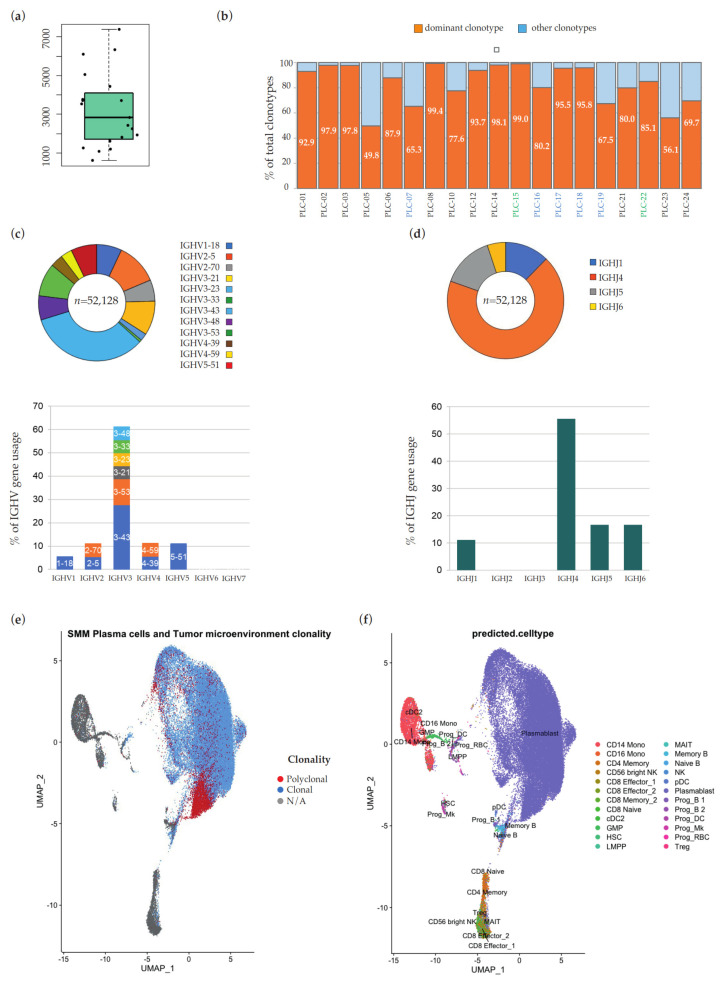
(**a**) Distribution of the number of barcodes estimated to be associated with cells that express targeted V(D)J transcripts in the 19 patients’ samples. (**b**) Percentage of barcodes associated with the dominant clonotype and with all other clonotypes, respectively, in the 19 patients’ samples. On the *x*-axis, IDs of MGUS samples are in blue, IDs of SMM samples are in black and IDs of MM samples are in green. VH (**c**) and JH (**d**) gene usage in 18/19 MM samples with clonal heavy chain rearrangements. The histograms show the sample-level rate of use of each IgH variable region gene family on total of clonal rearrangements. Within each V-family, discrete bands represent each of the individual genes, as indicated. Above each histogram, the representativeness of relative IGH genes/families is plotted at single-cell-level, as number of associated cellular barcodes. (**e**,**f**) UMAP dimension reduction of all the barcodes sequenced from samples obtained after CD138-based magnetic beads positive selection. In (**e**), light blue dots represent barcodes associated with the dominant clonotype of each sample, red dots barcodes associated with other rearrangements, and grey dots barcodes with unrearranged Ig heavy and light chain loci. In (**f**), dots are color-coded according to transcriptome-based cell type assignment, as indicated in the legend.

We purified CD138^+^ BM PCs with magnetic beads separation in 19 samples from 18 patients affected by MGUS (6), SMM (11), and MM (1). We analyzed a total of 61,022 barcodes estimated to be associated with cells that express targeted V(D)J transcripts, with an average of 3212 per sample (range: 606–7379) (Figure 1a). A dominant clonotype, i.e., one that was over-represented in each sample PC population, was easily identified in each sample. On average, 83.65% of V(D)J rearranged cells (range: 49.79–99.44% in each sample) belonged to the dominant clonotype (Figure 1b); in absolute numbers, the 19 clonotypes accounted for 53,935 of these 61,022 barcodes (53,935/61,022 = 88.39%). As expected, the most abundant IGHV family was IGHV3, with IGHV3-43 as the most represented gene, and IGHJ4 the prevalent IGHJ family (Figure 1c, d, respectively). In CellRanger output, we also found 8806 barcodes not associated with cells expressing targeted V(D)J transcripts (Figure 1e). According to automated transcriptome-based cell type assignment, these cells with unrearranged Ig heavy and light chain loci are components of the BM immune microenvironment (i.e., T cells, monocytes, and NK cells, detailed in Figure 1f), and the presence of their transcriptome in the 5′ gene expression libraries reflects unwanted contamination of the positive selection of CD138^+^ cells.

**Figure 2:**
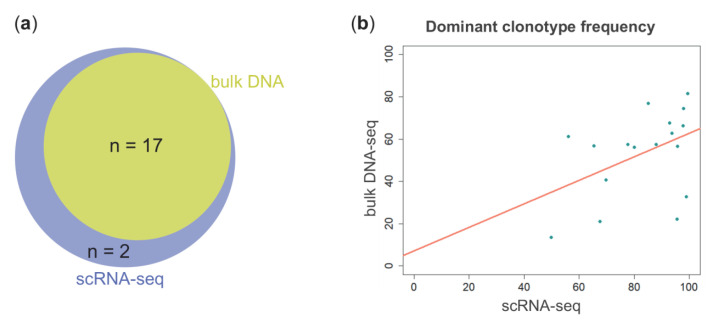
(**a**) Venn diagram showing the number of unique and shared clonotypes between scRNA-seq (blue circle) and bulk DNA (yellow circle) analyses. (**b**) Scatterplot of the percentage frequencies of the 17 dominant clonotypes in (**a**) as determined by scRNA-seq (*x* axis) and bulk DNA-seq (*y* axis) analyses.

We then compared scRNA-seq-based IGH analysis results with those obtained by applying the EuroClonality-NGS Working Group standard operating procedure for two-step Ig NGS-based marker identification, starting from genomic DNA of CD138^+^ cells. The V(D)J rearranged sequences of dominant clonotypes identified by the two methods were identical in 17/19 samples (Figure 2a, Table 1). Relative clonal fractions were heterogeneous, very moderately correlated inter-assay (Pearson’s product-moment correlation: R^2^ = 0.19; *p* value = 0.08), and constantly higher in scRNA-seq analysis (Figure 2b).

Dominant IGH rearrangement sequences determined by scRNA-seq and amplicon-based NGS from bulk DNA shared an exact match, supporting the robustness of the single cell-based approach. However, in 6/17 cases the V gene recognized as the most similar to the one involved in the patient-specific rearrangement by Vidjil and CellRanger was not the same (Table 1). This can be explained by slight differences in alignment among the two algorithms, made more likely by the low degree of homology of these genes with the germline repertoire due to their somatic hypermutation.

**Figure 3:**
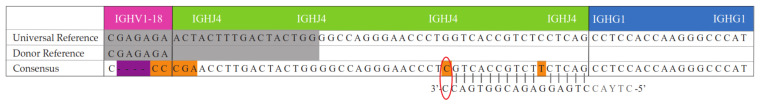
Alignment between the DNA sequence of EuroClonality-NGS protocol’s reverse primers IGH-J-A-1 and IGH-J-A-2 (written in 3′-to-5′ direction) and the rearranged sequence of the dominant clonotype (“Consensus”) as displayed by Loupe V(D)J browser for patient sample PLC-14. Nucleotides highlighted in orange in the Consensus sequence represent mutated positions compared with the germline IGHJ4 gene (’Universal Reference”). The gray background indicates where the sequences align to the reference. The purple background corresponds to a nucleotide deletion. The red oval indicates the mutated nucleotide causing a misannealing of input DNA with the 3’ end of both reverse primers used in the first-step PCR, and thus prevents PCR priming. Nucleotides indicated in grey in the primers’ sequence belong to the intron separating the J segment of the rearranged V-region and the C-region sequence. This intron is removed after transcription by RNA splicing joining the V-region exon to the C-region sequence, and for this reason, this portion of the reverse primers does not match with the consensus sequence of mature RNA transcript.

Two patients showed a discordant rearrangement between DNA-based NGS and scRNA-seq. For patient sample PLC-14, V(D)J analysis from bulk DNA identified only one clonotype corresponding to an unproductive rearrangement due to stop codons (IGHV3-23*01/IGHD3-9*01/IGHJ5*02) in 78% of reads. Conversely, scRNA-seq detected a productive rearrangement involving IGHV1-18 and IGHJ4 genes in 98.08% of barcodes. Each of these V(D)J sequences is exclusive to one type of approach, and there is no trace of it in the other. The failure of scRNA-seq to detect the unproductive rearrangement is expected and attributable to the degradation of the transcript containing premature translation-termination codons by the nonsense-mediated decay (NMD) mechanism. A mutation in the J gene involved in the productive rearrangement, on the other hand, is in all likelihood at the origin of its refractoriness to amplification by the EuroClonality-NGS protocol. The mutated nucleotide, in fact, causes a misannealing of input DNA with the 3′ end of both reverse primers used in the first-step PCR, and mismatches in this position are known to be detrimental to PCR priming (Figure 3).

Furthermore, our analysis from bulk DNA was unable to identify any productive clonotype in patient sample PLC-16, where only one dominant unproductive rearrangement was detected (IGHV3-11*05/IGHD3-3*01/IGHJ5*02, 29.97% of reads). ScRNA-seq of this sample found one light chain-only dominant clonotype (IGKV1-33/IGKJ3; 79% of reads). This was consistent with serum immunofixation electrophoresis results for this patient, showing a ĸ light-chain myeloma.

**Figure 4:**
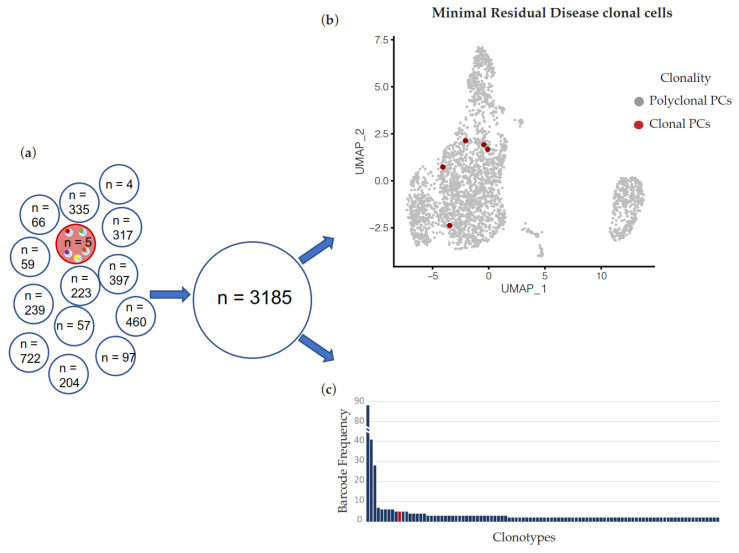
(**a**) In silico dilution of reads from 5 barcodes associated with the dominant clonotype of patient’s sample PLC-10 (red) into reads from 3180 barcodes of 13 different samples. (**b**) UMAP dimension reduction of the simulated sample. Red dots represent barcodes associated with PLC-10-specific V(D)J rearrangement. (**c**) Bar plot of clonotypes’ abundance in the virtual fastq file generated in (**a**) and analyzed by Loupe V(D)J Browser. Clonotypes after the 100th rank according to the barcode frequency are not plotted. The red bar represents the clonotype made up of the five barcodes sharing the myeloma-specific V(D)J rearrangement of PLC-10. The presence of the top-ranked clonotypes displayed at the left of the red bar is dependent on the procedure followed for the generation of the virtual sample, and is compatible with the composition of the V(D)J repertoire observable in a normal sample [17].

Our results therefore suggest that scRNA-seq can accurately identify dominant clonotypes in PC dyscrasias at diagnosis. We next asked if the same methodology can be applied to remission samples after treatment, i.e., if scRNA-seq can identify few residual cells of a known clonotype and therefore be a suitable methodology for transcriptomic characterization of MRD+ cells. To this end, we informatically generated a virtual patient sample where 5 known clonal PCs were admixed with 3180 cells with a polyclonal V(D)J sequence. Remarkably, the tool successfully identified all 5 cells belonging to the MRD clonotype within the polyclonal background (Figure 4).

## 3. Discussion

The tumor cell-specific rearrangement in the immunoglobulin V(D)J gene region (particularly at the IGH locus) is already exploited as a target by NGS (both amplicon- and capture-based [18]) or allele specific oligonucleotide (ASO) PCR. Here, the identification of the clonotypic rearrangement is particularly worthwhile also in routine clinical practice, as it represents an extremely specific molecular marker that is useful for monitoring the disease burden along its various stages. Indeed, MRD evaluation is proving more and more useful due to the impact that the depth of the response has on the outcome, and it could also act as a rapidly assessable surrogate trial endpoint, thus bridging the increasing delay between drug development and approval.

Overall, our findings argue that scRNA-seq provides robust and accurate data to derive the sequence of such clonal V(D)J rearrangements in single-cells. Specifically, in our study cohort, this approach proved even more effective in identifying clonal productive Ig rearrangements than the V(D)J analysis from bulk DNA, which in two cases failed. The data emerging from scRNA-seq could in both cases be exploited to overcome the limitations demonstrated by the conventional method. In particular, scRNA-seq can overcome false negative results due to DNA mispriming. Furthermore, the comprehensive DNA-based genotyping of the B cell receptor requires a high amount of starting DNA and a high number of PCRs, which can be a limiting factor in some circumstances, while analysis of few thousand cells is feasible in most cases. On the other hand, it is worth underlining that in MM, where the V(D)J rearrangements are expected to be productive, the Ig marker screening by scRNA-seq does not present some limitations which, on the contrary, may be a limiting factor in other B neoplasms, such as acute lymphoblastic leukemia (ALL). Here, in fact, most of the rearrangements are unproductive, and therefore the Ig/TCR marker screening by RNA-seq, targeting productive transcripts, can be incomplete [19].

ScRNA-seq, in its standard version and even more in its various implementations (CITE-seq, scATAC-seq, etc.), is an approach capable of providing an unprecedented amount of information, but still extremely expensive and demanding at the level of data analysis. For these reasons, its application is far from widespread, but remains rather limited to research scenarios. To maximize the output of scRNA-seq analysis in MM, parallel to a 5′ gene expression library, the generation of a V(D)J enriched library from amplified cDNA of the same cells constitutes a worthwhile solution that requires no additional input material, involves low wet-lab and sequencing costs, and an extremely simple data analysis. Indeed, the validation proposed here is of paramount importance as it opens new avenues of investigation for approaches aimed at functional single-cell MRD studies, where residual cells after treatment could be identified based on their V(D)J sequence and further characterized for gene and surface protein expression. The simulation of a post-therapy sample that we conducted by in silico dilution of clonal tumor cells supports the feasibility of such a scRNA-seq in the MRD context. Overall, our studies align with the general view that the potential of NGS is currently under-utilized in translational applications in MM [20,21,22], while a comprehensive characterization of its heterogeneity, as well as the functional properties of residual cells after treatment, may favorably impact the outcome of patients.

## 4. Materials and Methods

### 4.1. Patients

The study was based on a series of 18 patients with PC dyscrasia admitted to our institution. Monoclonal gammopathy of undetermined significance (MGUS), smoldering MM (SMM), and MM were diagnosed according to the International Myeloma Working Group (IMWG) revised criteria [23]. The study was approved by the local Ethics Committee (Provision n. 575 dated 29 March 2018) and written informed consent was obtained from all of the patients involved in the study. The study was conducted according to good clinical practice and the ethical principles outlined in the Declaration of Helsinki. BM sampling was done at diagnosis, and repeated in a SMM patient at progression to symptomatic disease, totaling 19 samples. White blood cells were obtained from BM aspirates, after red cells lysis, and CD138^+^ PCs were isolated by an immunomagnetic method with anti-CD138 monoclonal antibodies (STEMCELL Technologies, Vancouver, BC, Canada).

### 4.2. DNA-Based Molecular Analysis of IGHV Rearrangement

For the purification of genomic DNA from CD138^+^ PCs, we used the AllPrep DNA/RNA/miRNA isolation kit (Qiagen, Hilden, Germany), following the manufacturer’s protocol. We searched for clonal IGHV rearrangements by applying the EuroClonality-NGS Working Group standard operating procedure for two-step Ig NGS-based marker identification [Version 1.0 (11 June 2019)] using the IGH V-J set of primers. Libraries were pooled and sequenced using 250 bp paired-end runs on a MiSeq instrument (Illumina) to an average of 31.96 Mbp per sample.

Fastq files were uploaded on the Vidjil [16] web application for the analysis of high-throughput sequencing reads on IGH locus.

### 4.3. Single-Cell V(D)J Analysis

Fresh cells isolated via CD138^+^ magnetic bead separation were processed according to 10x Genomics Chromium Single-cell 5′ Gene Expression workflow and, in parallel, Chromium Single-cell V(D)J Enrichment protocol. In particular, sample partitioning and molecular barcoding were done on the Chromium Controller (10x Genomics), where we loaded cellular suspensions together with the Single-cell 5′ Gel Beads on a Single-cell 5′ chip, in which gel beads in emulsion (GEM) generation took place. Each gel bead is functionalized with barcoded oligonucleotides that consists of: (i) an Illumina R1 sequence, (ii) a 16 bp 10x barcode to index GEMs, (iii) a 10 bp randomer to index molecules (unique molecular identifier, UMI), and (iv) a 13 nt template switch oligo (TSO). Reverse transcription (RT) of polyadenylated RNA transcripts took place using poly(dT) primers inside each GEM, after which cDNAs (each containing a UMI and shared 10x barcode per GEM (cell), and ending with a TSO at the 3′ end) were pooled for amplification and library construction in bulk. Specifically, from the amplified cDNA of each sample, we prepared in parallel a 5′ gene expression library and a V(D)J enriched library. The latter was obtained after enrichment of 10x barcoded, full-length V(D)J segments via PCR amplification with primers specific to Ig constant regions. Generated libraries were combined according to Illumina specifications and paired-end sequenced (2 × 150 bp) on Illumina NovaSeq platform to a depth of ~150,000 and 15,000 reads/cell for 5′ gene expression and V(D)J enriched libraries, respectively.

We used the Cell Ranger software (10x Genomics) to process scRNA-seq data. In particular, *mkfastq* command allowed raw data demultiplexing, then *count* and *vdj* were used for transcriptome and V(D)J raw data alignment to the reference genome and gene count matrix generation, respectively. Output cloupe files and vloupe files (these latter containing clonotypes and CDR3 sequences deriving from paired clonotype calling) were overlaid for an integrated analysis by Loupe Browser and Loupe V(D)J Browser. In particular, Loupe VDJ Browser was used to explore the clonality and diversity of the B cell receptor repertoire at the single-cell level. For the performance evaluation of this immunoinformatics analysis, we generated an in silico dilution of reads from 5 barcodes associated with the dominant clonotype of a patient’s sample into reads from 3180 barcodes of 13 different samples. In parallel, samples were analyzed through the Seurat pipeline of analysis [24]. In detail, we initially performed a quality check assessment removing those cells with less than 200 and more than 3000 expressed features. The first threshold allowed us to remove possible cell-free mRNA, the second one to exclude doublets. Moreover, we also removed died/dying cells by filtering out cells expressing more than 5% of mitochondrial genes. Then, to better define the possible transcriptomic differences between each sample, we removed immunoglobulin related genes from the expression matrix. Finally, the 19 samples were integrated through the *findintegrationanchors* and *intergratedata* Seurat functions [24]. Once the integration was completed, the whole data set was processed using the Doubletfinder tool [25]: only cells defined as “singlets” were retained for further analysis. The entire dataset was Log normalized and scaled, and principal components analysis was performed. Automated cell assignment was performed anchoring our dataset to the already published bone marrow annotated atlas by Hao et al. [24]. The Uniform Manifold Approximation and Projection for Dimension Reduction (UMAP) was performed by the UMAP-learn algorithm [26].

## Figures and Tables

**Table 1:** Comparison of V(D)J clonal rearrangements at IGH locus identified by scRNA-seq and amplicon-based NGS from bulk DNA in the 17/19 samples in which the two methods gave matching results.

Patient ID	scRNA-Seq			Amplicon-Based NGS from Bulk DNA			Common CDR3
	IGHV	IGHJ	Proportion	IGHV	IGHJ	Proportion	
PLC-01	IGHV3-48	IGHJ4	92.90%	IGHV3-30*09	IGHJ4*02	67.55%	CARDSYEDYVYW
PLC-02	IGHV3-21	IGHJ4	97.93%	IGHV3-21*01	IGHJ4*02	74.39%	CARYQLDAVAGKWGHYFDYW
PLC-03	IGHV3-43	IGHJ5	97.77%	IGHV3-9*01	IGHJ4*02	66.23%	CAKARLPLVGGLDSW
PLC-05	IGHV3-43	IGHJ6	49.79%	IGHV3-9*01	IGHJ6*02	13.62%	CTRVIGSGASCYDCYYHGMDVW
PLC-06	IGHV3-53	IGHJ5	87.91%	IGHV3-53*01	IGHJ4*02	57.58%	CARGLTAPGFPLDSW
PLC-07	IGHV3-23	IGHJ6	65.26%	IGHV3-23*01	IGHJ6*01	56.92%	CAKGRADCTDGVCYRRYGMDVW
PLC-08	IGHV3-43	IGHJ4	99.44%	IGHV3-43*01	IGHJ4*02	81.44%	CVKGQGGYTYGGFDCW
PLC-10	IGHV5-51	IGHJ4	77.63%	IGHV5-51*01	IGHJ4*02	57.39%	CARTNWPYYFDHW
PLC-12	IGHV3-43	IGHJ4	93.73%	IGHV3-9*01	IGHJ4*02	62.86%	CARDRYQLIIYYFDRW
PLC-15	IGHV3-43	IGHJ4	98.97%	IGHV3-9*01	IGHJ4*02	32.77%	CAKDVRYGYGSTQSAGFDYW
PLC-17	IGHV4-39	IGHJ4	95.53%	IGHV4-39*07	IGHJ4*02	22.10%	CARDKTTMTFSSPIFDYW
PLC-18	IGHV2-5	IGHJ1	95.78%	IGHV2-5*02	IGHJ1*01	56.58%	CAHSGSMWSGYAGTEYFQHW
PLC-19	IGHV4-59	IGHJ4	67.50%	IGHV4-59*01	IGHJ4*02	21.08%	CARAGDYDLLLLDYW
PLC-21	IGHV5-51	IGHJ6	80.02%	IGHV5-51*03	IGHJ6*03	56.06%	CARLPQGGYYYMDVW
PLC-22	IGHV3-53	IGHJ5	85.10%	IGHV3-53*01	IGHJ4*02	77.01%	CARGLTAPGFPLDSW
PLC-23	IGHV3-33	IGHJ1	56.11%	IGHV3-30-3*02	IGHJ1*01	61.13%	CAFAIGADGEYFQHW
PLC-24	IGHV2-70	IGHJ4	69.68%	IGHV2-70*01	IGHJ4*02	40.67%	CARGASETQVAMSTAELYFFDSW

## Data Availability

Data are available upon request to the corresponding author.
